# Genome sequencing and protein domain annotations of Korean Hanwoo cattle identify Hanwoo-specific immunity-related and other novel genes

**DOI:** 10.1186/s12863-018-0623-x

**Published:** 2018-05-29

**Authors:** Kelsey Caetano-Anolles, Kwondo Kim, Woori Kwak, Samsun Sung, Heebal Kim, Bong-Hwan Choi, Dajeong Lim

**Affiliations:** 1 Interdisciplinary Program in Bioinformatics, Seoul National University, Kwan-ak St. 599, Kwan-ak Gu, Seoul, 151-741 Republic of Korea; 2 Department of Agricultural Biotechnology and Research Institute for Agriculture and Life Sciences, Seoul National University, Seoul, 151-921 Republic of Korea; 3 CHO&KIM genomics, Main Bldg. #514, SNU Research Park, Seoul National University Mt.4-2, NakSeoungDae, Gwanakgu, Seoul, 151-919 Republic of Korea; 4 Animal Genomics & Bioinformatics Division, National Institute of Animal Science, RDA, 77 Chuksan-gil, Kwonsun-gu, Suwon, 441-706 Republic of Korea

**Keywords:** Cattle, Hanwoo, Genome sequencing, Protein domain, Unaligned read assembly, DNA-Seq

## Abstract

**Background:**

Identification of genetic mechanisms and idiosyncrasies at the breed-level can provide valuable information for potential use in evolutionary studies, medical applications, and breeding of selective traits. Here, we analyzed genomic data collected from 136 Korean Native cattle, known as Hanwoo, using advanced statistical methods.

**Results:**

Results revealed Hanwoo-specific protein domains which were largely characterized by immunoglobulin function. Furthermore, domain interactions of novel Hanwoo-specific genes reveal additional links to immunity. Novel Hanwoo-specific genes linked to muscle and other functions were identified, including protein domains with functions related to energy, fat storage, and muscle function that may provide insight into the mechanisms behind Hanwoo cattle’s uniquely high percentage of intramuscular fat and fat marbling.

**Conclusion:**

The identification of Hanwoo-specific genes linked to immunity are potentially useful for future medical research and selective breeding. The significant genomic variations identified here can crucially identify genetic novelties that are arising from useful adaptations. These results will allow future researchers to compare and classify breeds, identify important genetic markers, and develop breeding strategies to further improve significant traits.

**Electronic supplementary material:**

The online version of this article (10.1186/s12863-018-0623-x) contains supplementary material, which is available to authorized users.

## Background

Hanwoo is a Korean native taurine breed of cattle that has been around since 2000 BC. Although their original primary purpose was to serve as farming and transportation cattle, the rapid growth of the Korean economy that occurred in the 1960’s and its associated food demands led to this breed being used as a main source of meat [1]. Since then, the demand for this product in Korea has skyrocketed. This is due to the high percentage of fat marbling in Hanwoo meat, a characteristic that is unique to the breed. Hanwoo loin muscles have approximately 24% intramuscular fat content [2]. The quality and price of meat is often determined by fat marbling. Consequently, one of the main goals of the meat production industry worldwide is to increase the incidence of this trait [2]. Given this focus, several studies have investigated gene expression patterns with the primary goal of determining which genes are responsible for Hanwoo-specific high fat concentration [3–7].

Here we gathered genomic data from 136 Hanwoo cattle that we analyzed using advanced statistical methods. We show that investigation of the genome of this unique set of cattle individuals with the general goal of identifying breed-level idiosyncrasies can provide valuable information for potential use in evolutionary studies, medical applications, and breeding of selective traits. The goal is to enhance our understanding of characteristics of beef cattle breeds with unique adaptations and beneficial traits that have not yet been well elucidated. This would make it possible to selectively breed for these traits in other breeds of cattle worldwide to improve meat quality and revolutionize the field of meat production.

## Methods

### Alignment of unaligned reads for the detection for novel genes using the Hanwoo whole genome

Blood samples for whole genome sequencing were obtained from 136 Korean beef cattle (Hanwoo) individuals reared at the Hanwoo Improvement Center of the National Agricultural Cooperative Federation (Seosan, Chungnam, Korea). Indexed shotgun paired-end (PE) libraries with 500 bp average length inserts were generated from these samples using the TruSeq Nano DNA Library Prep Kit (Illumina, USA) following the standard Illumina sample-preparation protocol. Briefly, 200 ng of gDNA was fragmented using a Covaris M220 focused-ultrasonicator (Woburn, MA, USA) to produce fragments with a median size of ~ 500 bp. The fragmented DNA was subjected to end repair, A-tailing, and indexed adapter ligation (~ 125 bp adapter). Adapter-ligated DNA of 550 to 650 bp in length was amplified using PCR for 8 cycles. The size-selected libraries were analyzed using the Agilent 2100 Bioanalyzer (Agilent Technologies) to determine the size distribution and to check for adapter contamination. The resulting libraries were sequenced using the Illumina HiSeq 2500 (2x125bp paired-end sequences) and NextSeq500 (2x150bp paired-end sequences) Next-Gen sequencers.

**Fig. 1:**
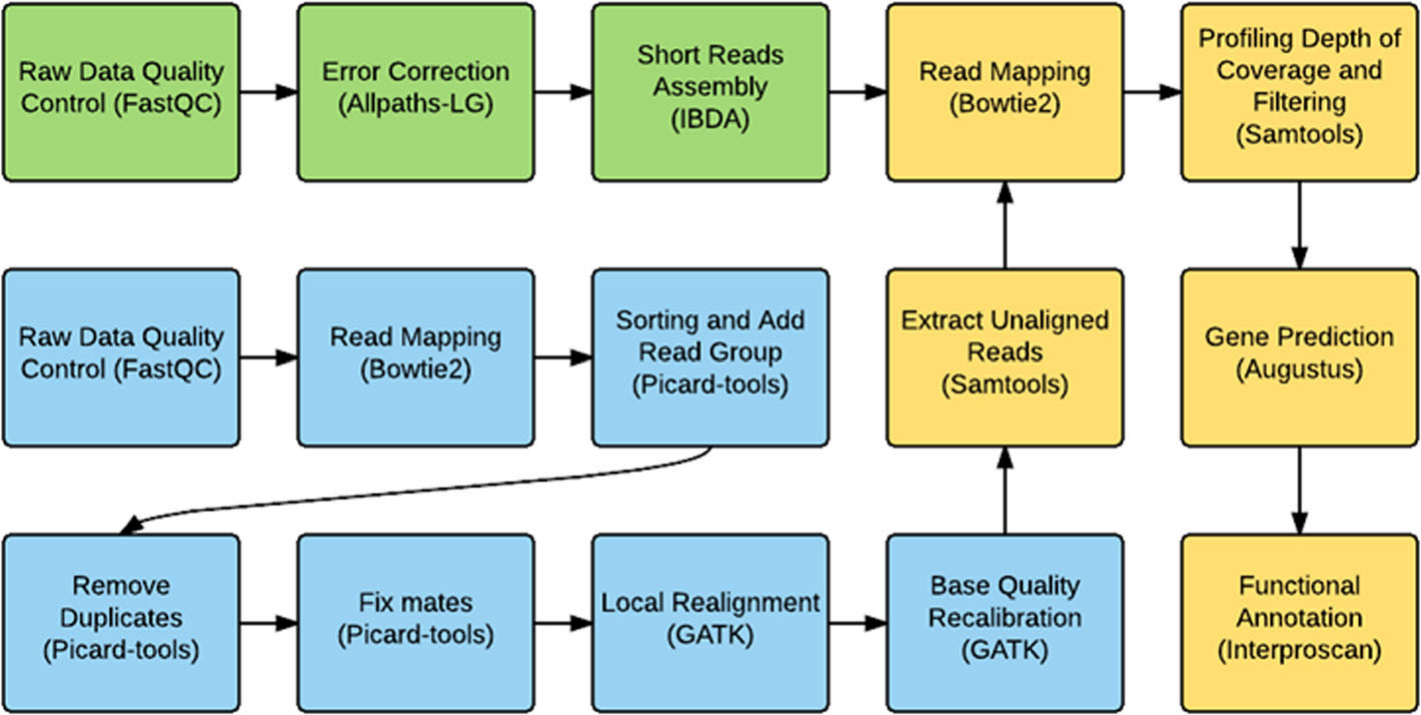
Detailed unaligned read assembly pipeline. Green squares represent the first stage of analysis- assembly of scaffold-level genome; blue squares represent the second stage of analysis- extraction of unaligned reads; yellow squares represent the third and final stage of analysis- gene prediction and functional annotation

**Fig. 2:**
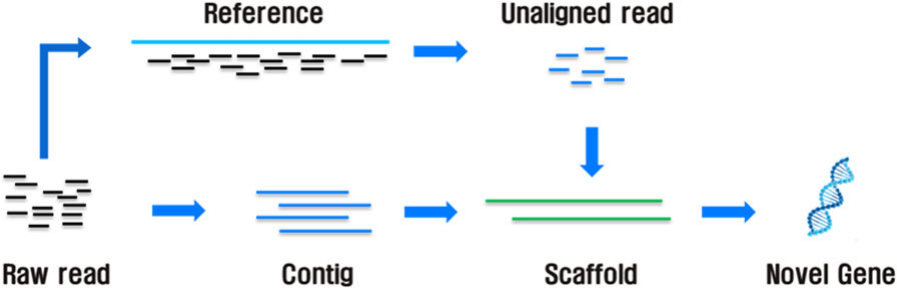
Simplified pipeline of unaligned read assembly

The bioinformatics pipeline used in this study is described in Figs. 1 and 2. Quality control for per-base quality of reads and removal of potential adaptor sequences was performed using fastQC v0.11.4 [8] and Trimmomatic v0.36 [9] software (seed mismatches:2, palindrome clip threshold:30, simple clip threshold:10, LEADING:10, TRAILING:10, MINLEN:80), respectively. Then, high-quality sequence reads were mapped to the *Bos taurus* reference genome (UMD 3.1) using Bowtie2.2.6 [10] with default settings in order to extract unaligned reads. Removal of duplicate reads was performed using Picard (ver 1.06) and indexing, sorting, and unaligned read extraction was performed using Samtools v1.3.1 [11]. GATK v3.4.46 [12–14] was used for local realignment and recalibration of the alignment (blue boxes on the pipeline figure; Fig. 1). A summary of sequencing data is provided in Additional file 1: Table S1.

Since we are interested in information originating from the sample itself and not detected from the reference sequence, we created an assembled genome at the scaffold level to discover whether unaligned reads actually constitute functional units (genes) on their own genome. This scaffold was created from one randomly selected sample from our pool of samples. The Broad Institute’s stand-alone ALLPATHS-LG fragment read error correction module [15, 16] was used for error correction as a precursor to de novo assembly. De novo assembly was performed using an Iterative De Bruijn Assembler of Uneven Depth (IDBA_UD: [17, 18], an iterative De Bruijn graph de novo assembler for short reads sequencing data that utilizes paired-end reads to assemble highly uneven low-depth regions. This tool is useful for optimizing the length gap problem and iterating different K-mer length (green boxes on the pipeline figure; Fig. 1).

For unaligned read alignments, we extracted reads for each sample that was not aligned to the reference genome. Using the extracted unaligned reads (blue boxes on the pipeline figure; Fig. 1) and the assembled scaffold-level genome (green boxes on the pipeline figure; Fig. 1) of each sample, alignment of unaligned reads to the scaffold was carried out using Bowtie2 (remapping). The identified remapped sequences throughout the sequence were assumed to represent Hanwoo-specific sequences. These resulting regions constitute regions that are distinctive from the reference. We performed depth profiling to diminish the possibility of false positives. We identified scaffolds containing locations meeting our depth cutoff of 10× (an arbitrary cutoff selected for result filtering), and used the collected scaffolds for gene prediction using the gene prediction program Augustus 3.1.0. Out of the resulting 614 predicted genes, we extracted protein sequences covered by unaligned reads with at least depth of 10×.

The resulting total of 283 protein sequences were cross-referenced against the Pfam database of protein families (pfam.xfam.org; [19]) using the protein domain detection program InterProScan-5.15-54.0 in order to identify protein domains affiliated with those areas of the genome. In order to assign meaning and infer the function of these domains, we searched for these identified domains within DOMINE (http://domine.utdallas.edu/cgi-bin/Domine), a database of known and predicted protein domain interactions [20, 21]. Using Interpro [22], we obtained GO (Gene Ontology) Cellular Component (CC), Molecular Function (MF), and Biological Process (BP) terms for each individual domain [23]. Next, gene ontology results were summarized and visualized with the online tool REVIGO (http://revigo.irb.hr; [24]) to better interpret our results. Next, using REVIGO’s *Interactive Graph* tool [24] and exporting results into the Cytoscape software package [25], we created a graph-based visualization of the identified terms for each GO category.

Using the above described methodologies and annotations we were able to align and map genome sequences as well as predict genes that may be related to Hanwoo-specific characteristics.

## Results and discussion

### Research objectives and genome build summary

Our main research objectives included: (1) Assembling and mapping unaligned reads in order to identify and predict genes in Hanwoo cattle; (2) Cross-referencing results against a comprehensive protein domain database in order to identify protein domains affiliated with those areas of the genome; and (3) Mining the uncovered genes and associated domains to identify important gene functions and networks involved in positive traits.

A summary of representative reference genome builds via short read assembly is presented in Table 1. We mapped unaligned reads against the reference genome and extracted information to a depth of 10× (meaning that each base was sequenced an average of 10 times). We predicted a total of 614 gene regions using scaffolds containing locations higher than depth of 10×. Of the 614 genes, 283 genes were covered by unaligned reads with at least depth of 10×.

**Table 1:** Summary of the results of representative reference genome build via short read assembly (> = 1 kb)

		Base pairs	Percent (%)
Number of scaffolds		295,265	100
Residue counts	A	701,475,984	29.24
	C	498,799,879	20.79
	G	498,441,379	20.78
	T	692,999,844	28.89
	N	7,063,142	0.29
	Total	2,398,780,228	100
Sequence lengths	Minimum	1000	
	Maximum	136,625	
	Average	8124.16	
	N50	13,528	

Cross-referencing of protein sequences from the 283 genes against the Pfam database identified associated protein domains covering a total of 168 scaffolds. Overall, 311 Pfam protein domains were identified when using data filtered for sequences with an average mapped base depth coverage of less than 10×. These numbers suggest that there was more than one affiliated domain identified for some gene regions. Due to space limitations, Table 2 lists significantly identified (*E*- value <1XE-100) Pfam protein family domain analysis results. An extended list of significantly identified Pfam domains with *E*-value <1E-40 is presented in Additional file 2: Table S2.

**Table 2:** Significantly identified (*E*- value <1XE-100) Pfam protein family domain analysis results

Gene name	Length	Source	Accession	Description	Start	Stop	*E*-value
scaffold_2197.g59.t1	581	Pfam	PF00063	Myosin head (motor domain)	30	575	5.60E-207
scaffold_1285.g30.t1	417	Pfam	PF15718	Domain of unknown function (DUF4673)	116	412	4.50E-154
scaffold_6851.g129.t1	391	Pfam	PF03028	Dynein heavy chain and region D6 of dynein motor	2	390	1.50E-120
scaffold_13817.g209.t1	758	Pfam	PF01403	Sema domain	59	467	2.30E-117
scaffold_29068.g344.t1	348	Pfam	PF16021	Programmed cell death protein 7	33	344	3.00E-114
scaffold_15941.g224.t1	887	Pfam	PF04849	HAP1 N-terminal conserved region	1	249	2.60E-108
scaffold_5769.g113.t1	246	Pfam	PF00244	14–3-3 protein	5	238	3.60E-107
scaffold_1936.g56.t1	564	Pfam	PF08235	LNS2 (Lipin/Ned1/Smp2)	300	525	1.30E-104

### Hanwoo-specific genes linked to immunity

A number of domains were largely characterized by immune system function. Selected immune system-related genes are shown in Table 3. Six of the seven domains shown are associated to the immunoglobulin function, while the remaining domain is associated with the interferon group of signaling proteins, which is crucial for the immune system response as well.

**Table 3:** Selected immune system-related genes and affiliated protein domains

Gene name	Length	Source	Accession	Description	Start	Stop	*E*-value
scaffold_2520.g67.t1	1075	Pfam	PF13895	Immunoglobulin domain	425	491	5.40E-09
scaffold_19370.g263.t1	508	Pfam	PF07679	Immunoglobulin I-set domain	381	454	8.30E-07
scaffold_8624.g151.t1	512	Pfam	PF13895	Immunoglobulin domain	10	73	3.90E-08
scaffold_14147.g214.t1	159	Pfam	PF07679	Immunoglobulin I-set domain	27	112	3.40E-24
scaffold_13817.g209.t1	758	Pfam	PF00047	Immunoglobulin domain	550	628	5.10E-09
scaffold_5779.g114.t1	142	Pfam	PF07679	Immunoglobulin I-set domain	35	70	6.20E-07
scaffold_46987.g437.t1	460	Pfam	PF09294	Interferon-alpha/beta receptor, fibronectin type III	44	142	1.20E-17

The interferon-alpha/beta receptor is a cell surface receptor made up of one chain with two subunits, IFNAR1 and IFNAR2. The interferon receptors have antiviral, antiproliferative, and immunomodulatory functions, as well as being highly involved in pregnancy [26, 27]. Interferon-τ, a type I interferon, has been shown to prevent a return to ovarian cyclicity after conception to ensure the continuation of the pregnancy in ruminant ungulate species; this interferon appears to be the main factor responsible for prevention of degradation of the corpus luteum [28, 29].

In addition to these reproductive roles, this receptor is responsible for binding type 1 interferons interferon–α and –β and activating the JAK-STAT signaling pathway, which is associated with DNA-transcription and the expression of genes related to immunity, proliferation, and differentiation, among others [30]. The JAK-STAT pathway has primary functions related to immunity. In fact, drug therapies that aim to turn down the immune response of the body and modulate host responses to disease and infection target this pathway [31]. The expression of the interferon group of signaling proteins in our Hanwoo cattle samples suggests that Hanwoo may have breed-specific immune system functions that are not yet well understood.

Our analysis also identified associated protein domains which are largely characterized by the immunoglobulin function. These results are particularly salient given the significance of these kinds of results for medical research and selective breeding. The bovine immune system has been a topic of interest to researchers for quite some time now, mainly due to two reasons [32]. The first is that an understanding of the evolution and expression of mammalian immune system genes has important implications for human health. Bovine antibodies have been of particular interest, as they exhibit prophylactic and therapeutic properties in response to several human and animal infectious diseases [33–36]. Additionally, researchers have recently developed transgenic calves that produce human immunoglobulin, speaking to the incredible importance of cattle as model organisms for the study of human immunity and disease [37]. Secondly, understanding the molecular and genetic basis of immunity in cattle breeds can not only serve to further our understanding of the breeds, but also to provide genetic information which can be used for selective breeding in order to improve performance and survival of livestock. Immunity in cattle varies vastly by breed. For example, African cattle are known for their incredible resistance to tick and gastrointestinal parasite infestations, traits that have developed in response to thousands of years of evolution in the harsh environments of Africa. A particularly amazing adaptation is the resistance of several African breeds to trypanosomiasis, also known as sleeping sickness [38]. Identification of genes responsible for immunity and introduction of identified immunity-related genes in cattle breeds that are productive but highly susceptible to disease may improve their resistance, survival, and productivity. Understanding genetic features controlling these mechanisms will allow researchers to develop appropriate breeding strategies.

More generally, research in immunoglobulin genetics is particularly salient for several reasons. Although research into the genetic aspects of and expression of genes related to immunoglobulin has been widely conducted in humans and mice, research in this field is lacking when it comes to livestock breeds, particularly cattle. Information is still needed to complete previous information, including the number of available gene segments and gene families. This kind of information can be used in the future to study and create synthetic recombinant species-specific antibodies, which could be used to treat and prevent infectious diseases.

### Domain interactions of Hanwoo-specific genes reveal additional links to immunity

Additionally, more general consideration of significantly identified protein family domains from the Pfam database provided information needed to further understanding the breed-specific molecular mechanisms of Hanwoo cattle. Table 2 lists highly significantly identified (*E*- value <1XE-100) Pfam domains. In order to assign meaning and infer the function of these domains, which include several not well understood but highly significant protein domains, we searched for these identified domains within DOMINE (http://domine.utdallas.edu/cgi-bin/Domine), a database of known and predicted protein domain interactions [20, 21]. Among these, several interesting results reveal the genetic intricacies of the Hanwoo genome and its functions.

Several of the most significantly identified protein domains appear to be closely linked with immune system function, further supporting our previous findings. For example, the significantly identified Sema domain (*E*-value = 2.30E-117) appears to be primarily associated with immune system function. The Sema domain not only forms interactions with the Immunoglobulin domain, but also interacts with the Thrombospondin type 1 (TSP-1) domain, which has been shown to control immune regulation. Thrombospondin, an extremely large multi-domain glycoprotein, is crucial to certain mechanisms related to angiogenesis, cell proliferation, and immune responses [39] such as the chemotactic response to tissue damage and the facilitation of phagocytosis of damaged cells [40–42]. Mice deficient in TSP-1 are more susceptible to inflammation and injury, either as a side effect of drugs or as a result of gene activation [43–46]. Given the strong role of this protein domain in immunity, our identification of this pathway here once again confirms that there are unique functions of immunity at play operating specifically in the Hanwoo genome.

### Hanwoo-specific genes linked to muscle and other functions

Significantly identified protein domains with functions related to energy, fat storage, and muscle function may provide insight into the mechanisms behind Hanwoo cattle’s uniquely high percentage of intramuscular fat and fat marbling. For example, LNS2 (Lipin/Ned1/Smp2) domain, which includes Lipin, was significantly identified (*E*-value = 1.30E-104) in our data (Table 2). Lipin, encoded by the *Lpin1* gene, is a powerful gene which largely controls how the body produces, stores, and uses fat. Mice deficient in Lipin do not develop either diet-induced or genetic obesity [47]. Additionally, enhanced Lipin expression has been shown to promote adiposity in mice [48].

Additionally, the Myosin head (motor domain) protein domain, which is associated with muscle function, was significantly identified (*E*-value = 5.60E-207, Table 2). Myosin is a chief component of myofibril filaments, which are responsible for muscle contraction. Myosin also actively participates in the conversion of ATP chemical energy to mechanical energy through its interaction with Actin [49]. Additionally, the Dynein heavy chain and region D6 of the dynein motor domain and 14–3-3 protein domain were significantly identified (*E*-values = 1.50E-120,3.60E-107 respectively), both of which are also largely responsible for ATP energy conversion [50–52]. These results suggest that these proteins domains are those which are primarily responsible for providing energy to the muscle and possibly causing the breed-specific high percentage of intramuscular fat that is observed in Hanwoo cattle.

Several of the other identified domains, such as the HAP1 N-terminal conserved region domain, were found to lack interactions with any other domains and their specific roles in cattle have not been well established. As we learn more about these proteins and their functions in the future, we may be able to better interpret these results.

### Interpretation of gene ontology terms associated with the entire set of Pfam domains

As previously discussed, we were able to identify 311 Pfam domains mapping to 168 scaffolds not shared with common cattle. We then filtered that list and kept only the highest hits. Within that short list, we revealed high enrichment for muscle and immunology genes. However, this approach provides a very limited look at our results. Thus, we aimed to further explore Hanwoo-specific domains by analyzing the enrichment of functional categories associated with each individual domain of the entire list. Using Interpro [22], we obtained GO (Gene Ontology) Cellular Component (CC), Molecular Function (MF), and Biological Process (BP) terms for each individual domain [23]. Next, gene ontology results were summarized and visualized with the online tool REVIGO (http://revigo.irb.hr; [24] to better interpret our results. Tables 4, 5, and 6 summarize the BP, CC, and MF GO terms, respectively. REVIGO calculates “frequency” and “uniqueness” values, with *frequency* representing the proportion of the specified GO term within the entire *Bos taurus* species-specific Uniprot protein annotation database, and *uniqueness* determining within the inputted list whether a term is an outlier when compared semantically to the list as a whole [24].

**Table 4:** Summary of enriched Gene Ontology (GO) biological process (BP) terms among total identified Pfam protein family domains

term_ID	description	Frequency^a^	log10 *p*-value	Uniqueness^b^
GO:0008152	metabolic process	62.92%	−4.2076	0.974
GO:0007154	cell communication	28.75%	−5.9208	0.866
GO:0006139	nucleobase-containing compound metabolic process	28.16%	−31.0655	0.776
GO:0007165	signal transduction	26.76%	−18.9208	0.794
GO:0006810	transport	19.48%	−32.6383	0.765
GO:0006355	regulation of transcription, DNA-templated	14.27%	−8.9586	0.608
GO:0006464	cellular protein modification process	14.26%	−11.7212	0.62
GO:0007186	G-protein coupled receptor signaling pathway	8.87%	−25.4318	0.803
GO:0006508	proteolyis	7.74%	−32.6778	0.741
GO:0006811	ion transport	7.05%	−12.2076	0.744
GO:0055114	oxidation-reduction process	6.85%	−11.699	0.831
GO:0055085	transmembrane transport	6.54%	−32.6383	0.714
GO:0016192	vesicle-mediated transport	4.60%	−30.7447	0.785
GO:0006886	intracelular protein transport	3.09%	−30.7447	0.792
GO:0016567	protein ubiquitination	2.40%	−14.3468	0.673
GO:0006820	anion transport	2.07%	−61.2147	0.769
GO:0006457	protein folding	1.00%	−19.9208	0.736
GO:0007018	microtubule-based movement	1.00%	−45.6021	0.843
GO:0035023	regulation of Rho protein signal transduction	0.92%	−11.1675	0.832
GO:0016573	histone acetylation	0.60%	−51.3372	0.635
GO:0043401	steroid hormone mediated signaling pathway	0.46%	−28.6383	0.839
GO:0045454	cell redox homeostasis	0.40%	−27.0269	0.865
GO:0000413	protein peptidyl-prolyl isomerization	0.24%	−19.9208	0.709
GO:0006400	tRNA modification	0.20%	−34.7447	0.692
GO:0018149	peptide cross-linking	0.17%	−13.4318	0.731
GO:0007099	centriole replication	0.08%	− 153.347	0.832
GO:0009396	folic acid-containing compound biosynthetic process	0.05%	−11.699	0.786

^a^*Frequency* represents the proportion of the specified GO term within the entire *Bos Taurus* species-specific Uniprot protein annotation database. Higher frequencies represent more general and common terms, while terms with a lower frequency are rare and specific

^b^*Uniqueness* represents whether a term is an outlier when compared semantically to the list as a whole

**Table 5:** Summary of enriched Gene Ontology (GO) cellular component (CC) terms among total identified Pfam protein family domains

term_ID	description	Frequency^a^	log10 *p*-value	Uniqueness^b^
GO:0005622	intracellular	63.18%	−4.2076	0.875
GO:0016020	membrane	47.23%	−5.7959	0.872
GO:0016021	integral component of membrane	29.77%	−10.8239	0.832
GO:0005634	nucleus	27.70%	−10.6576	0.688
GO:0005739	mitochondrion	9.22%	−12.5686	0.636
GO:0005740	mitochondrial envelope	3.09%	−32.3565	0.576
GO:0031012	extracellular matrix	2.00%	−15	0.77
GO:0016459	myosin complex	0.38%	− 206.252	0.589
GO:0030286	dynein complex	0.20%	−45.6021	0.594

^a^*Frequency* represents the proportion of the specified GO term within the entire *Bos Taurus* species-specific Uniprot protein annotation database. Higher frequencies represent more general and common terms, while terms with a lower frequency are rare and specific

^b^*Uniqueness* represents whether a term is an outlier when compared semantically to the list as a whole

**Table 6:** Summary of enriched Gene Ontology (GO) molecular function (MF) terms among total identified Pfam protein family domains

term_ID	description	Frequency^a^	log10 p-value	Uniqueness^b^
GO:0003700	sequence-specific DNA binding transcription factor activity	5.30%	−10.6576	0.958
GO:0003712	transcription cofactor activity	1.78%	−39.8861	0.957
GO:0003824	catalytic activity	37.22%	−13.4559	0.972
GO:0004871	signal transducer activity	11.94%	−25.4318	0.927
GO:0004930	G-protein coupled receptor activity	7.87%	−27.6198	0.925
GO:0005089	Rho guanyl-nucleotide exchange factor activity	0.48%	−11.699	0.956
GO:0005216	ion channel activity	2.49%	−13.9208	0.896
GO:0015075	ion transmembrane transporter activity	5.28%	−5.7959	0.895
GO:0016773	phosphotransferase activity, alcohol group as acceptor	4.60%	−58.7447	0.771
GO:0004672	protein kinase activity	3.87%	− 14.1308	0.772
GO:0043015	gamma-tubulin binding	0.10%	−25.8539	0.878
GO:0008017	microtubule binding	1.06%	−16.4815	0.848
GO:0019001	guanyl nucleotide binding	2.48%	−25.4318	0.846
GO:0005509	calcium ion binding	3.94%	−19.0655	0.863
GO:0005515	protein binding	26.71%	−7.3665	0.92
GO:0004488	methylenetetrahydrofolate dehydrogenase (NADP+) activity	0.02%	−13.4559	0.878
GO:0003755	peptidyl-prolyl cis-trans isomerase activity	0.26%	−19.9208	0.876
GO:0003777	microtubule motor activity	0.51%	−45.6021	0.814
GO:0005544	calcium-dependent phospholipid binding	0.17%	−19.0655	0.844
GO:0042802	identical protein binding	4.77%	−25.8539	0.878
GO:0031683	G-protein beta/gamma-subunit complex binding	0.16%	−25.4318	0.883
GO:0043565	sequence-specific DNA binding	4.31%	−10.6576	0.855
GO:0016787	hydrolase activity	15.05%	−7.8861	0.841
GO:0008484	sulfuric ester hydrolase activity	0.11%	−31.7447	0.832
GO:0004181	metallocarboxypeptidase activity	0.15%	−32.6778	0.809
GO:0004222	metalloendopeptidase activity	0.79%	−15	0.79
GO:0019901	protein kinase binding	1.80%	−7.2007	0.885
GO:0008479	queuine tRNA-ribosyltransferase activity	0.02%	−34.7447	0.849
GO:0003676	nucleic acid binding	21.33%	−8.6021	0.849
GO:0005102	receptor binding	6.56%	−62.1871	0.875
GO:0004402	histone acetyltransferase activity	0.24%	−51.3372	0.812
GO:0003677	DNA binding	10.28%	−9.5376	0.843
GO:0008408	3′-5′ exonuclease activity	0.18%	−31.0655	0.827
GO:0016746	transferase activity, transferring acyl groups	1.42%	−30.9208	0.805
GO:0003723	RNA binding	7.68%	−5.9208	0.847
GO:0046872	metal ion binding	20.96%	−4.5376	0.845
GO:0004550	nucleoside diphosphate kinase activity	0.14%	−55.585	0.816
GO:0008236	serine-type peptidase activity	1.32%	−7.1739	0.793
GO:0008270	zinc ion binding	6.73%	−4.2076	0.856
GO:0004129	cytochrome-c oxidase activity	0.43%	−12.5686	0.809
GO:0003924	GTPase activity	1.03%	−25.4318	0.806
GO:0005524	ATP binding	8.83%	−14.1308	0.751
GO:0000166	nucleotide binding	14.40%	−7.3565	0.823
GO:0003810	protein-glutamine gamma-glutamyltransferase activity	0.07%	−14.7696	0.823
GO:0005543	phospholipid binding	1.33%	−16.6021	0.829
GO:0035091	phosphatidylinositol binding	0.84%	−17	0.829

^a^*Frequency* represents the proportion of the specified GO term within the entire *Bos Taurus* species-specific Uniprot protein annotation database. Higher frequencies represent more general and common terms, while terms with a lower frequency are rare and specific

^b^*Uniqueness* represents whether a term is an outlier when compared semantically to the list as a whole

**Fig. 3:**
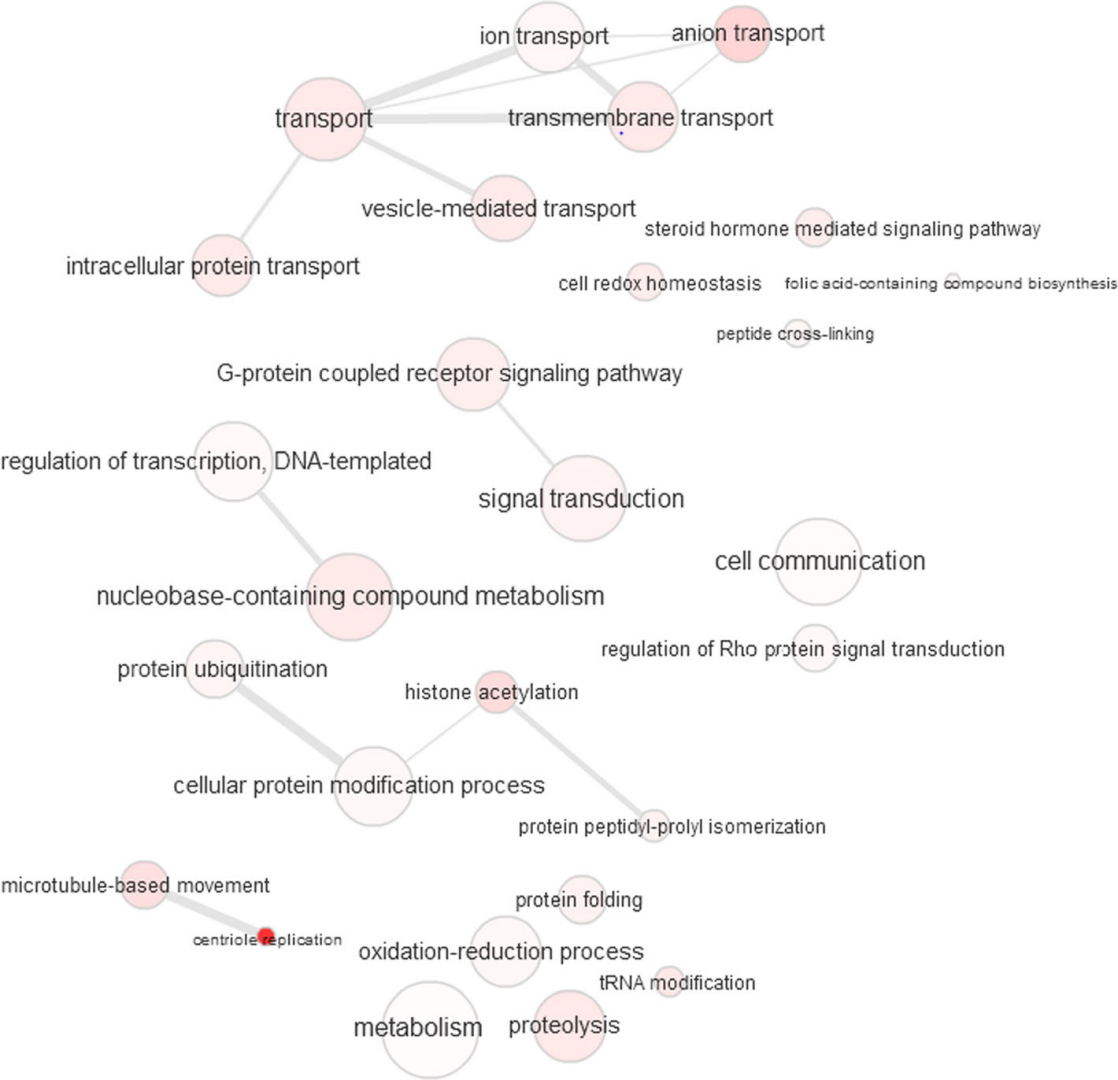
Visualization of significantly identified Gene Ontology (GO) Biological Process (BP) terms. The radius of the bubbles represents the generality of the specified term (a small bubble implies higher specificity). The *p*-value of each GO term is represented by the color shading of each bubble (darker colors representing higher significance). The edges between the nodes of our graph (GO terms) represent the top 3% strongest pairwise similarities between terms

**Fig. 4:**
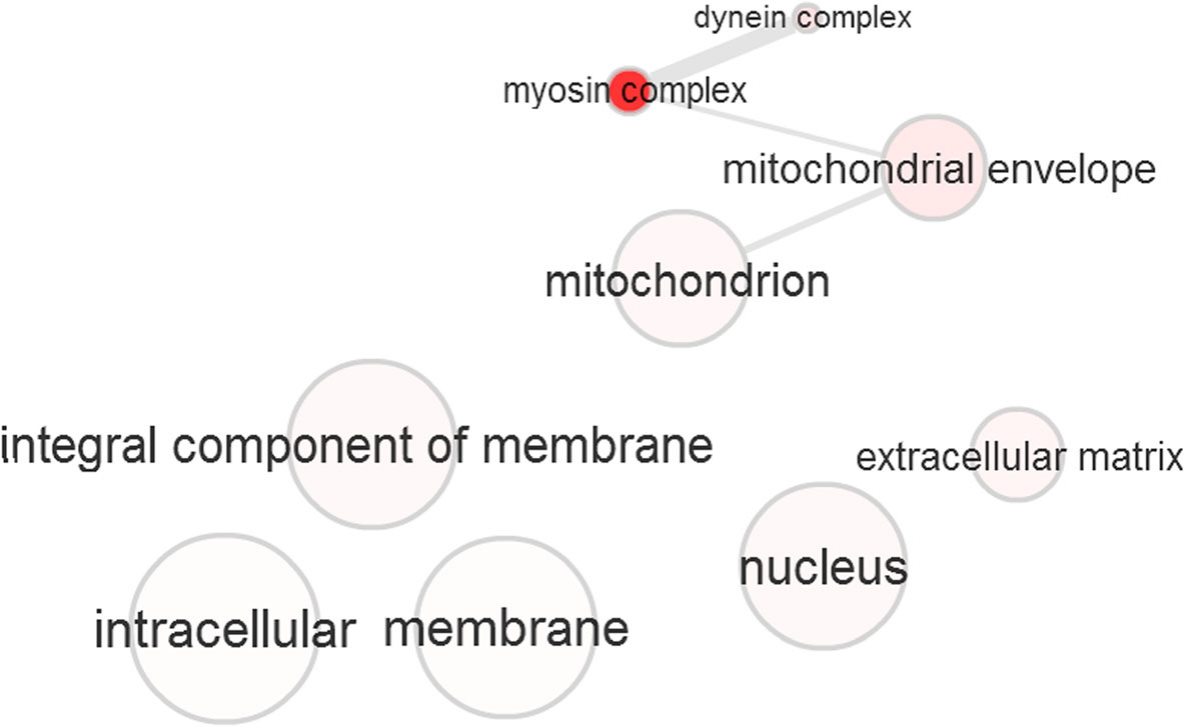
Visualization of significantly identified Gene Ontology (GO) Cellular Component (CC) terms. The radius of the bubbles represents the generality of the specified term (a small bubble implies higher specificity). The p-value of each GO term is represented by the color shading of each bubble (darker colors representing higher significance). The edges between the nodes of our graph (GO terms) represent the top 3% strongest pairwise similarities between terms

**Fig. 5:**
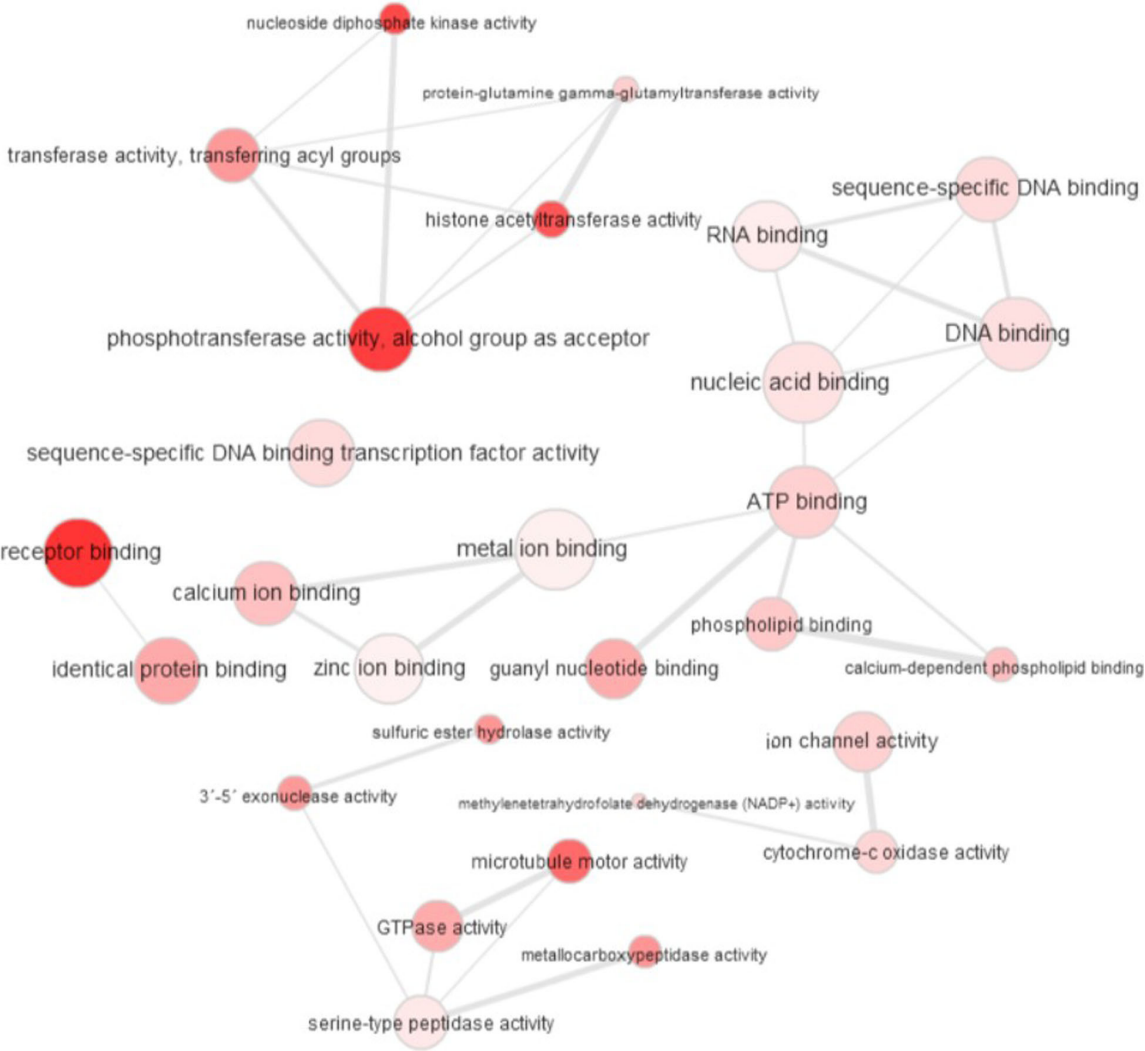
Visualization of significantly identified Gene Ontology (GO) Molecular Function (MF) terms. The radius of the bubbles represents the generality of the specified term (a small bubble implies higher specificity). The p-value of each GO term is represented by the color shading of each bubble (darker colors representing higher significance). The edges between the nodes of our graph (GO terms) represent the top 3% strongest pairwise similarities between terms

Next, using REVIGO’s *Interactive Graph* tool [24] and exporting results into the Cytoscape software package [25], we created a graph-based visualization of the identified terms for each GO category. Figures 3, 4, and 5 display visualizations of BP, CC, and MF GO terms, respectively. The radius of the bubbles represents the generality of the specified term; a small bubble implies higher specificity. The *p*-value of each GO term is represented by the color shading of each bubble, with darker colors representing higher significance. The edges between the nodes of our graph (GO terms) represent the top 3% strongest pairwise similarities between terms [24].

The BP GO term visualization (Fig. 3) can be characterized by a large number of un-connected solo terms and shows a large diversity of biological processes being affected, meaning that a large rewiring of functionality is embedded in the new genes acquired by Hanwoo cattle. Note that the most significant term is the most specific, ‘centriole replication’, which is also connected to the general term ‘microtubule-based movement’; dynein (significantly identified from our data) moves along microtubules, so this term may reflect the biological processes responsible for dynein’s role in ATP energy conversion. This is quite unique and unexpected, since it signals an important role of cell division [53]. The second group of more significant terms are less specific but all related to transport, particularly ‘anion transport’, which may be associated with ATP energetics. Another uniqueness is the steroid hormone mediated signaling pathway. Sex steroid hormones play a critical role in the regulation of muscle, muscle strength, and growth and maintenance of muscle mass [54]. While identification of this GO term most likely can be attributed to the aforementioned relationship between steroid hormones and muscle development, as a result of the breed-specific unique high-fat muscle development, it may also be due to the practices under which Hanwoo are reared in order to enhance the natural fat marbling in their meat, such as feeding time and diet. For example, cattle are fed a high-concentration grain diet as opposed to grass-feeding [55]. Diet has been shown to have an effect on steroid hormones [56], which may also in part explain the identification of this GO term here.

The CC GO term visualization (Fig. 4) can be characterized by a single connected group consisting of four terms: dynein complex, myosin complex, mitochondrial envelope, and mitochondrion. As previously mentioned, the Myosin Head and Dynein heavy chain protein domains were found significantly identified in our results- both of which participate in the conversion of ATP chemical energy to mechanical energy and serve crucial functions for muscle function. The connectivity of these nodes within our network visualization signifies that these two components work together and are potentially significant in Hanwoo-specific characteristics, such as their high percentage of intramuscular fat. The rest of the terms are generic, independent CC terms that include nucleus and membrane.

The MF GO term visualization (Fig. 5) can be characterized by high connectivity, with the most significant values grouped together. Microtubule motor activity, another microtubule function related term, was also identified at the molecular function level, once again suggesting ATP energetics at play. A unique feature of this visualization, compared to the BP and CC visualizations, is the presence of 4 unconnected graphs as opposed to many unconnected terms or a single connected group. The first group features solely terms related to binding. This group contains the following terms: Sequence-specific DNA binding, DNA binding, RNA binding, Nucleic acid binding, ATP binding, Phospholipid binding, Calcium-dependent phospholipid binding, Guanyl nucleotide binding, Metal ion binding, Zinc ion binding, and Calcium ion binding. The second group consists of three connected terms: Ion Channel activity, Methylenetetrahydrofolate dehydrogenase (NADP+) activity, and Cytochrome-c oxidase activity. The third group consists of six connected terms: Sulfuric ester hydrolase activity, 3′-5′ exonuclease activity, Microtubule motor activity, GTPase activity, Serine-type peptidase activity, and Metallocarboxypeptidase activity.

The fourth and final group consists of 5 terms related to the activity of transferases: Nucleoside diphosphate kinase activity, Transferase activity, transferring acyl groups, Protein-glutamine gamma-glutamyltransferase activity, Histone acetyltransferase activity, and Phosphotransferase activity, alcohol group as acceptor. Transferases are enzymes which are responsible for catalyzation of the transfer of certain functional groups from one molecule to another. They are essential for countless biochemical processes throughout the body. In cattle specifically, it has been shown that the activity of transferases is critical for embryo development [57]. The expression of genes with transferase activity function varies between abnormal and normal pregnancies [58, 59]. Therefore, the expression of these transferase GO terms may be due to their role in healthy pregnancy and development. However, interestingly, results of previous studies have demonstrated a correlation between certain transferase activity genes, such as GPAT1 and ATGL, and intramuscular fat content in Korean Cattle [60]. These previously identified results, when taken along with the comparatively high expression and connectivity of GO terms related to transferase activity, suggests that there may be unique mechanisms of transferase activity in Hanwoo cattle which influences their development and may perhaps be a factor impacting their species-specific high percentage of intramuscular fat.

## Conclusions

The information unearthed from the comparison of breeds and identification of genetic variation in this study will be invaluable for future research on the molecular determinants that have been bred in Hanwoo cattle. Results revealed Hanwoo-specific protein domains which were largely characterized by immunoglobulin function. Furthermore, domain interactions of Hanwoo-specific genes reveal additional links to immunity. Hanwoo-specific genes linked to muscle and other functions were identified, including protein domains with functions related to energy, fat storage, and muscle function that may provide insight into the mechanisms behind Hanwoo cattle’s uniquely high percentage of intramuscular fat and fat marbling. Analyzing the whole Hanwoo genome and reporting significant genomic variations is crucial to identifying genetic novelties that are arising from useful adaptations. Similarly, such analysis will allow future researchers to compare and classify breeds, identify important genetic markers, and develop breeding strategies to further improve traits of economic value and biological significance.

## Additional files

Additional file 1: **Table S1.** Summary of sequencing data (DOCX 28 kb)

Additional file 2: **Table S2.** Significantly identified (*E*- value <1XE-40) Pfam protein family domain analysis results. (DOCX 17 kb)

Additional file 3: FASTA sequences for scaffolds which have locations with depth > 10×. (XLSX 9907 kb)

Additional file 4: Protein sequences which have locations with depth > 10×. (XLSX 101 kb)

## Abbreviations

BP: Biological process
CC: Cellular component
GO: Gene ontology
MF: Molecular function
