# Supplementary material for: Genome sequencing and protein domain annotations of Korean Hanwoo cattle identify Hanwoo-specific immunity-related and other novel genes
Source: BMC Genet. 2018 May 29;19:37. doi: 10.1186/s12863-018-0623-x (PMC5975384; doi:10.1186/s12863-018-0623-x)
Supplement: Supplementary file 1 — Table S1. Summary of sequencing data (DOCX 28 kb) [file 12863_2018_623_MOESM1_ESM.docx]

Table S1. Summary of sequencing data

| **Sample** | **Total Reads** | **Overall alignment rate (%)** | **Genome coverage (%)** | **Average read depth** | **Scaffold coverage (%)** | **Average read depth** |
| --- | --- | --- | --- | --- | --- | --- |
| 12007 | 592,041,182 | 89.63% | 98.89% | 25.82 | 6.91 | 0.17 |
| 12042 | 497,379,490 | 88.98% | 99.08% | 21.49 | 6.51 | 0.16 |
| 12059 | 500,517,846 | 90.61% | 98.86% | 22.03 | 6.03 | 0.15 |
| 13065 | 474,578,512 | 86.46% | 98.84% | 20.06 | 7.61 | 0.16 |
| 13303 | 553,468,696 | 86.12% | 99.07% | 23.30 | 8.51 | 0.19 |
| 24078 | 563,146,494 | 87.20% | 98.86% | 23.81 | 8.01 | 0.19 |
| 27034 | 456,454,022 | 84.89% | 98.89% | 19.16 | 7.47 | 0.16 |
| 27214 | 528,406,446 | 89.85% | 99.00% | 22.83 | 6.53 | 0.16 |
| 29012 | 505,454,830 | 85.54% | 98.89% | 21.25 | 7.98 | 0.17 |
| 29058 | 530,625,258 | 87.63% | 98.91% | 22.85 | 7.68 | 0.17 |
| KPN036 | 529,128,398 | 84.85% | 99.00% | 22.19 | 11.10 | 0.22 |
| KPN056 | 517,937,406 | 84.92% | 99.01% | 21.72 | 10.76 | 0.21 |
| KPN059 | 515,627,468 | 85.56% | 98.99% | 21.60 | 11.07 | 0.21 |
| KPN083 | 517,691,260 | 83.32% | 99.00% | 20.92 | 11.01 | 0.21 |
| KPN103 | 498,206,088 | 87.93% | 98.98% | 21.65 | 8.81 | 0.18 |
| KPN104 | 514,280,348 | 87.42% | 98.99% | 22.19 | 9.32 | 0.19 |
| KPN108 | 516,619,182 | 88.96% | 98.98% | 22.69 | 8.73 | 0.17 |
| KPN116 | 500,063,696 | 87.56% | 99.00% | 21.36 | 9.15 | 0.19 |
| KPN123 | 492,769,206 | 92.52% | 98.97% | 22.00 | 5.48 | 0.13 |
| KPN124 | 471,831,944 | 92.39% | 98.97% | 21.08 | 5.44 | 0.14 |
| KPN131 | 474,728,892 | 92.34% | 98.98% | 21.30 | 5.83 | 0.14 |
| KPN136 | 465,722,102 | 91.69% | 99.02% | 20.72 | 6.07 | 0.14 |
| KPN137 | 463,939,802 | 91.65% | 98.98% | 20.76 | 6.40 | 0.14 |
| KPN141 | 485,644,124 | 89.29% | 98.97% | 21.20 | 8.47 | 0.17 |
| KPN144 | 469,797,966 | 92.02% | 98.97% | 20.87 | 6.13 | 0.14 |
| KPN147 | 567,529,396 | 92.39% | 99.01% | 25.12 | 6.64 | 0.16 |
| KPN151 | 493,490,882 | 89.45% | 98.94% | 21.74 | 8.67 | 0.18 |
| KPN184 | 528,674,862 | 86.83% | 99.01% | 22.54 | 10.55 | 0.21 |
| KPN195 | 543,585,540 | 87.88% | 99.00% | 23.10 | 9.88 | 0.20 |
| KPN200 | 504,950,630 | 88.18% | 98.94% | 21.69 | 9.20 | 0.18 |
| KPN218 | 515,643,382 | 85.04% | 98.95% | 20.56 | 10.68 | 0.22 |
| KPN219 | 519,312,026 | 86.25% | 98.99% | 22.19 | 12.00 | 0.22 |
| KPN227 | 509,852,918 | 87.70% | 98.92% | 21.80 | 9.95 | 0.19 |
| KPN229 | 577,038,954 | 87.77% | 98.99% | 24.23 | 10.30 | 0.21 |
| KPN506 | 536,152,376 | 88.23% | 99.04% | 22.52 | 9.47 | 0.20 |
| KPN546 | 567,007,714 | 88.47% | 99.04% | 24.11 | 8.02 | 0.19 |
| KPN565 | 510,374,794 | 88.86% | 99.06% | 21.71 | 7.54 | 0.17 |
| KPN593 | 505,797,030 | 88.24% | 98.98% | 21.16 | 7.66 | 0.18 |
| KPN673 | 496,578,220 | 88.13% | 99.04% | 21.58 | 8.95 | 0.18 |
| KPN712 | 502,546,936 | 84.88% | 98.91% | 20.14 | 9.31 | 0.21 |
| KPN716 | 602,970,002 | 87.41% | 99.04% | 25.28 | 8.84 | 0.21 |
| KPN725 | 546,064,758 | 88.14% | 99.06% | 23.22 | 8.10 | 0.19 |
| KPN768 | 515,643,594 | 91.48% | 99.01% | 22.02 | 5.57 | 0.15 |
| KPN769 | 576,870,886 | 88.17% | 99.06% | 25.05 | 8.86 | 0.21 |
| KPN775 | 528,817,950 | 91.80% | 99.01% | 23.19 | 5.82 | 0.15 |
| TN1409D3056 | 478,782,034 | 98.64% | 99.02% | 21.53 | 3.67 | 0.21 |
| TN1409D3057 | 568,620,742 | 98.52% | 99.04% | 24.90 | 3.57 | 0.21 |
| TN1409D3058 | 613,243,352 | 98.41% | 99.06% | 26.00 | 3.58 | 0.24 |
| TN1409D3059 | 633,019,312 | 98.53% | 99.06% | 27.68 | 3.59 | 0.23 |
| TN1409D3060 | 499,170,506 | 98.40% | 99.04% | 21.80 | 3.55 | 0.20 |
| TN1409D3061 | 635,883,144 | 98.37% | 99.06% | 27.88 | 3.54 | 0.23 |
| TN1409D3062 | 561,693,610 | 98.31% | 99.06% | 24.48 | 3.45 | 0.20 |
| TN1409D3063 | 593,908,658 | 98.44% | 99.05% | 25.95 | 3.53 | 0.22 |
| TN1409D3064 | 610,877,150 | 98.50% | 99.04% | 26.66 | 3.58 | 0.23 |
| TN1409D3065 | 535,767,258 | 98.50% | 99.05% | 23.50 | 3.62 | 0.21 |
| TN1409D3066 | 596,322,070 | 98.42% | 99.06% | 26.03 | 3.54 | 0.22 |
| TN1409D3067 | 640,125,998 | 98.10% | 99.05% | 26.94 | 3.37 | 0.19 |
| TN1409D3068 | 520,370,720 | 98.51% | 99.01% | 22.88 | 3.52 | 0.20 |
| TN1409D3069 | 592,619,730 | 98.50% | 99.05% | 26.14 | 3.53 | 0.22 |
| TN1409D3070 | 478,587,124 | 98.59% | 98.95% | 20.20 | 3.60 | 0.24 |
| TN1409D3071 | 536,112,062 | 98.75% | 99.01% | 23.01 | 3.52 | 0.25 |
| TN1409D3072 | 592,678,262 | 98.43% | 99.03% | 26.13 | 3.53 | 0.22 |
| TN1409D3073 | 589,273,640 | 98.50% | 99.03% | 25.90 | 3.51 | 0.22 |
| TN1409D3074 | 630,236,090 | 98.53% | 99.04% | 27.65 | 3.57 | 0.24 |
| TN1409D3075 | 603,142,558 | 98.44% | 99.05% | 26.44 | 3.29 | 0.21 |
| TN1409D3076 | 633,135,604 | 98.54% | 99.06% | 28.08 | 3.46 | 0.23 |
| TN1409D3077 | 608,111,964 | 98.46% | 99.06% | 26.93 | 3.59 | 0.23 |
| TN1409D3078 | 497,348,604 | 98.61% | 99.02% | 22.02 | 3.60 | 0.21 |
| TN1409D3079 | 543,194,082 | 98.60% | 99.06% | 24.08 | 3.59 | 0.22 |
| TN1409D3080 | 534,623,976 | 98.66% | 98.99% | 22.47 | 3.65 | 0.26 |
| TN1409D3081 | 598,671,608 | 98.14% | 99.05% | 25.31 | 3.54 | 0.20 |
| TN1409D3082 | 597,168,756 | 98.60% | 99.05% | 26.41 | 3.60 | 0.22 |
| TN1409D3083 | 594,162,360 | 98.48% | 99.04% | 26.36 | 3.51 | 0.23 |
| TN1409D3084 | 527,355,974 | 98.61% | 99.02% | 22.42 | 3.43 | 0.25 |
| TN1409D3085 | 508,827,104 | 98.52% | 98.99% | 21.39 | 3.46 | 0.23 |
| TN1409D3086 | 552,543,996 | 98.56% | 99.03% | 25.26 | 3.64 | 0.22 |
| TN1409D3087 | 599,411,652 | 98.55% | 99.04% | 26.99 | 3.49 | 0.22 |
| TN1409D3088 | 475,587,232 | 98.79% | 99.00% | 21.16 | 3.68 | 0.24 |
| TN1409D3089 | 396,863,234 | 98.86% | 98.97% | 17.57 | 3.62 | 0.21 |
| TN1409D3090 | 570,377,254 | 98.57% | 99.04% | 25.54 | 3.57 | 0.22 |
| TN1409D3091 | 453,014,248 | 98.71% | 98.99% | 19.49 | 3.59 | 0.25 |
| TN1409D3092 | 496,178,924 | 98.60% | 99.01% | 22.33 | 3.69 | 0.21 |
| TN1409D3093 | 601,033,008 | 98.28% | 99.05% | 25.57 | 3.62 | 0.20 |
| TN1409D3094 | 608,792,050 | 98.47% | 99.06% | 26.88 | 3.49 | 0.22 |
| TN1409D3095 | 660,184,602 | 98.26% | 99.08% | 29.06 | 3.48 | 0.22 |
| TN1409D3096 | 586,514,578 | 98.45% | 99.07% | 25.56 | 3.62 | 0.22 |
| TN1409D3097 | 648,032,770 | 98.23% | 99.08% | 28.43 | 3.54 | 0.22 |
| TN1409D3098 | 608,009,350 | 98.46% | 99.03% | 26.89 | 3.52 | 0.21 |
| TN1409D3099 | 518,056,934 | 98.47% | 99.01% | 21.57 | 3.46 | 0.24 |
| TN1409D3100 | 470,489,226 | 98.46% | 98.99% | 19.80 | 3.54 | 0.27 |
| TN1409D3101 | 503,785,960 | 98.56% | 99.01% | 21.36 | 3.60 | 0.25 |
| TN1409D3102 | 515,056,400 | 98.69% | 99.02% | 22.01 | 3.50 | 0.25 |
| TN1409D3103 | 535,028,580 | 98.59% | 99.01% | 22.44 | 3.57 | 0.25 |
| TN1409D3104 | 505,606,150 | 98.85% | 98.98% | 22.09 | 3.64 | 0.26 |
| TN1409D3105 | 554,832,976 | 98.65% | 98.99% | 23.70 | 3.42 | 0.25 |
| TN1409D3106 | 517,539,740 | 98.70% | 99.02% | 22.13 | 3.49 | 0.24 |
| TN1409D3107 | 521,099,244 | 98.69% | 99.00% | 22.67 | 3.53 | 0.26 |
| TN1409D3108 | 556,511,498 | 98.77% | 99.00% | 23.53 | 3.66 | 0.27 |
| TN1409D3109 | 551,083,512 | 98.76% | 99.02% | 24.19 | 3.61 | 0.25 |
| TN1409D3110 | 530,668,624 | 98.65% | 99.04% | 22.42 | 3.58 | 0.25 |
| TN1409D3111 | 523,349,164 | 98.74% | 99.03% | 22.80 | 3.46 | 0.24 |
| TN1409D3112 | 619,514,110 | 98.76% | 99.04% | 27.65 | 3.63 | 0.28 |
| TN1410D3741 | 544,322,048 | 98.75% | 98.95% | 23.26 | 3.67 | 0.25 |
| TN1410D3742 | 582,823,144 | 98.87% | 99.00% | 24.95 | 3.62 | 0.27 |
| TN1410D3744 | 573,736,638 | 98.73% | 99.00% | 24.26 | 3.45 | 0.25 |
| TN1410D3745 | 510,870,842 | 98.82% | 98.96% | 21.75 | 3.52 | 0.23 |
| TN1410D3746 | 514,908,570 | 98.85% | 98.98% | 21.95 | 3.43 | 0.23 |
| TN1410D3747 | 524,278,624 | 98.69% | 98.99% | 22.27 | 3.54 | 0.24 |
| TN1410D3748 | 576,486,340 | 98.83% | 99.00% | 24.31 | 3.75 | 0.27 |
| TN1410D3749 | 532,022,438 | 98.89% | 98.98% | 22.53 | 3.60 | 0.24 |
| TN1410D3750 | 563,008,884 | 98.72% | 98.97% | 23.52 | 3.44 | 0.25 |
| TN1410D3751 | 523,738,164 | 98.88% | 99.00% | 21.98 | 3.64 | 0.26 |
| TN1410D3752 | 578,166,338 | 98.96% | 98.98% | 24.44 | 3.78 | 0.28 |
| TN1410D3753 | 535,927,778 | 98.85% | 98.96% | 22.68 | 3.74 | 0.26 |
| TN1410D3754 | 553,875,676 | 98.81% | 98.99% | 23.09 | 3.56 | 0.26 |
| TN1410D3755 | 479,762,938 | 98.89% | 98.99% | 21.19 | 3.75 | 0.24 |
| TN1410D3756 | 466,509,610 | 98.72% | 98.99% | 20.57 | 3.67 | 0.23 |
| TN1410D3757 | 512,198,054 | 98.92% | 99.00% | 22.68 | 3.67 | 0.24 |
| TN1410D3758 | 591,323,290 | 98.74% | 99.03% | 25.31 | 3.42 | 0.23 |
| TN1410D3759 | 593,668,618 | 98.84% | 99.02% | 25.86 | 3.57 | 0.25 |
| TN1410D3760 | 516,617,062 | 98.85% | 98.99% | 22.01 | 3.65 | 0.24 |
| TN1410D3761 | 591,873,780 | 98.81% | 99.01% | 25.51 | 3.46 | 0.25 |
| TN1410D3762 | 496,726,936 | 98.64% | 99.01% | 20.88 | 3.58 | 0.24 |
| TN1410D3763 | 554,419,388 | 98.85% | 99.01% | 23.50 | 3.63 | 0.25 |
| TN1410D3764 | 522,921,070 | 98.81% | 98.99% | 22.14 | 3.49 | 0.23 |
| TN1410D3765 | 541,741,198 | 98.68% | 99.03% | 22.54 | 3.43 | 0.23 |
| TN1410D3766 | 545,970,064 | 98.77% | 99.01% | 22.94 | 3.58 | 0.25 |
| TN1410D3767 | 532,611,750 | 98.78% | 99.02% | 22.53 | 3.57 | 0.24 |
| TN1410D3768 | 480,040,420 | 98.73% | 98.96% | 20.34 | 3.59 | 0.23 |
| TN1410D3769 | 529,757,642 | 98.73% | 99.02% | 22.06 | 3.57 | 0.25 |
| TN1410D3770 | 553,061,170 | 98.83% | 99.01% | 23.25 | 3.37 | 0.23 |
| TN1410D3771 | 612,773,060 | 98.54% | 99.04% | 25.44 | 3.48 | 0.25 |
| TN1410D3772 | 563,181,006 | 98.78% | 99.02% | 23.66 | 3.58 | 0.25 |
| TN1410D3773 | 567,933,596 | 98.77% | 99.02% | 23.99 | 3.51 | 0.24 |
| TN1410D3879 | 548,492,566 | 98.64% | 99.02% | 22.72 | 3.57 | 0.24 |
| TN1411D3979 | 630,088,402 | 98.85% | 99.03% | 26.36 | 3.62 | 0.27 |
