# Supplementary material for: Genome sequencing and protein domain annotations of Korean Hanwoo cattle identify Hanwoo-specific immunity-related and other novel genes
Source: BMC Genet. 2018 May 29;19:37. doi: 10.1186/s12863-018-0623-x (PMC5975384; doi:10.1186/s12863-018-0623-x)
Supplement: Supplementary file 2 — Table S2. Significantly identified (E- value <1XE-40) Pfam protein family domain analysis results. (DOCX 17 kb) [file 12863_2018_623_MOESM2_ESM.docx]

Table S2. Significantly identified (E- value < 1XE-40) Pfam protein family domain analysis results

| **Gene name** | **Length** | **Source** | **Accession** | **Description** | **Start** | **Stop** | **E-value** |
| --- | --- | --- | --- | --- | --- | --- | --- |
| scaffold_2197.g59.t1 | 581 | Pfam | PF00063 | Myosin head (motor domain) | 30 | 575 | 5.60E-207 |
| scaffold_1285.g30.t1 | 417 | Pfam | PF15718 | Domain of unknown function (DUF4673) | 116 | 412 | 4.50E-154 |
| scaffold_6851.g129.t1 | 391 | Pfam | PF03028 | Dynein heavy chain and region D6 of dynein motor | 2 | 390 | 1.50E-120 |
| scaffold_13817.g209.t1 | 758 | Pfam | PF01403 | Sema domain | 59 | 467 | 2.30E-117 |
| scaffold_29068.g344.t1 | 348 | Pfam | PF16021 | Programmed cell death protein 7 | 33 | 344 | 3.00E-114 |
| scaffold_15941.g224.t1 | 887 | Pfam | PF04849 | HAP1 N-terminal conserved region | 1 | 249 | 2.60E-108 |
| scaffold_5769.g113.t1 | 246 | Pfam | PF00244 | 14-3-3 protein | 5 | 238 | 3.60E-107 |
| scaffold_1936.g56.t1 | 564 | Pfam | PF08235 | LNS2 (Lipin/Ned1/Smp2) | 300 | 525 | 1.30E-104 |
| scaffold_13665.g200.t1 | 671 | Pfam | PF08016 | Polycystin cation channel | 180 | 530 | 7.50E-93 |
| scaffold_117.g1.t1 | 383 | Pfam | PF15802 | DDB1- and CUL4-associated factor 17 | 100 | 288 | 4.70E-88 |
| scaffold_46558.g435.t1 | 330 | Pfam | PF07714 | Protein tyrosine kinase | 58 | 301 | 6.30E-88 |
| scaffold_7705.g140.t1 | 228 | Pfam | PF06027 | Solute carrier family 35 | 30 | 212 | 1.90E-85 |
| scaffold_163371.g598.t1 | 243 | Pfam | PF12774 | Hydrolytic ATP binding site of dynein motor region D1 | 9 | 136 | 9.20E-82 |
| scaffold_25421.g310.t1 | 251 | Pfam | PF13281 | Domain of unknown function (DUF4071) | 38 | 249 | 4.80E-80 |
| scaffold_1420.g33.t1 | 1490 | Pfam | PF00069 | Protein kinase domain | 729 | 1020 | 8.40E-73 |
| scaffold_9854.g164.t1 | 682 | Pfam | PF10456 | WASP-binding domain of Sorting nexin protein | 388 | 543 | 1.10E-70 |
| scaffold_15941.g224.t1 | 887 | Pfam | PF12448 | Kinesin associated protein | 310 | 478 | 4.40E-67 |
| scaffold_20989.g270.t1 | 150 | Pfam | PF16422 | Transcription factor COE1 DNA-binding domain | 1 | 119 | 1.70E-65 |
| scaffold_5796.g115.t1 | 876 | Pfam | PF14722 | Ki-ras-induced actin-interacting protein-IP3R-interacting domain | 150 | 300 | 6.50E-63 |
| scaffold_55928.g473.t1 | 249 | Pfam | PF00955 | HCO3- transporter family | 5 | 183 | 6.10E-62 |
| scaffold_16566.g234.t1 | 332 | Pfam | PF11822 | Domain of unknown function (DUF3342) | 147 | 299 | 2.70E-61 |
| scaffold_12733.g190.t1 | 1232 | Pfam | PF08623 | TATA-binding protein interacting (TIP20) | 1041 | 1202 | 1.70E-60 |
| scaffold_18391.g253.t1 | 156 | Pfam | PF15298 | AJAP1/PANP C-terminus | 30 | 133 | 3.20E-60 |
| scaffold_19619.g266.t1 | 806 | Pfam | PF00454 | Phosphatidylinositol 3- and 4-kinase | 254 | 467 | 1.80E-59 |
| scaffold_75312.g522.t1 | 89 | Pfam | PF12774 | Hydrolytic ATP binding site of dynein motor region D1 | 2 | 89 | 1.50E-58 |
| scaffold_66940.g503.t1 | 152 | Pfam | PF00334 | Nucleoside diphosphate kinase | 5 | 138 | 2.60E-56 |
| scaffold_2584.g69.t1 | 187 | Pfam | PF11841 | Domain of unknown function (DUF3361) | 20 | 149 | 3.40E-54 |
| scaffold_1531.g38.t1 | 1137 | Pfam | PF08214 | Histone acetylation protein | 948 | 1120 | 4.60E-52 |
| scaffold_32189.g371.t1 | 727 | Pfam | PF05729 | NACHT domain | 184 | 353 | 9.30E-51 |
| scaffold_12815.g191.t1 | 382 | Pfam | PF16212 | Phospholipid-translocating P-type ATPase C-terminal | 198 | 310 | 2.30E-49 |
| scaffold_17594.g246.t1 | 261 | Pfam | PF03028 | Dynein heavy chain and region D6 of dynein motor | 28 | 240 | 2.50E-46 |
| scaffold_16980.g238.t1 | 594 | Pfam | PF07714 | Protein tyrosine kinase | 380 | 503 | 1.20E-45 |
| scaffold_55806.g471.t1 | 235 | Pfam | PF01746 | tRNA (Guanine-1)-methyltransferase | 54 | 226 | 4.90E-43 |
| scaffold_19052.g259.t1 | 749 | Pfam | PF00092 | von Willebrand factor type A domain | 1 | 165 | 5.50E-42 |
| scaffold_5131.g105.t1 | 1007 | Pfam | PF12851 | Oxygenase domain of the 2OGFeDO superfamily | 903 | 1007 | 2.70E-41 |
